# GSK-3**β**: A Bifunctional Role in Cell Death Pathways

**DOI:** 10.1155/2012/930710

**Published:** 2012-05-21

**Authors:** Keith M. Jacobs, Sandeep R. Bhave, Daniel J. Ferraro, Jerry J. Jaboin, Dennis E. Hallahan, Dinesh Thotala

**Affiliations:** ^1^Department of Radiation Oncology, Washington University in St. Louis, St. Louis, MO 63108, USA; ^2^School of Medcine, Washington University in St. Louis, St. Louis, MO 63108, USA; ^3^Siteman Cancer Center, St. Louis, MO 63110, USA; ^4^Mallinckrodt Institute of Radiology, St. Louis, MO 63110, USA

## Abstract

Although glycogen synthase kinase-3 beta (GSK-3**β**) was originally named for its ability to phosphorylate glycogen synthase and regulate glucose metabolism, this multifunctional kinase is presently known to be a key regulator of a wide range of cellular functions. GSK-3**β** is involved in modulating a variety of functions including cell signaling, growth metabolism, and various transcription factors that determine the survival or death of the organism. Secondary to the role of GSK-3**β** in various diseases including Alzheimer's disease, inflammation, diabetes, and cancer, small molecule inhibitors of GSK-3**β** are gaining significant attention. This paper is primarily focused on addressing the bifunctional or conflicting roles of GSK-3**β** in both the promotion of cell survival and of apoptosis. GSK-3**β** has emerged as an important molecular target for drug development.

## 1. Introduction

Glycogen synthase kinase-3 is a ubiquitously expressed protein kinase that exists in two isoforms, *α* and *β*. Originally identified based on its role in glycogen biosynthesis based on its inactivating phosphorylation of glycogen synthase, it has since been found to regulate a myriad of functions through Wnt and other signaling pathways [1]. The two isoforms are strongly conserved within their kinase domain but differ greatly at the C-terminus, while the *α* isoform additionally contains a glycine-rich N-terminus extension [2]. Our paper will focus on the *β* isoform due to its more established role in cell survival and viability. Glycogen synthase kinase-3 beta (GSK-3*β*) is involved in the regulation of a wide range of cellular functions including differentiation, growth, proliferation motility, cell cycle progression, embryonic development, apoptosis, and insulin response [1–8]. It has emerged as an important regulator of neuronal, endothelial, hepatocyte, fibroblast, and astrocyte cell death in response to various stimuli [6, 7, 9].

**Figure 1:**
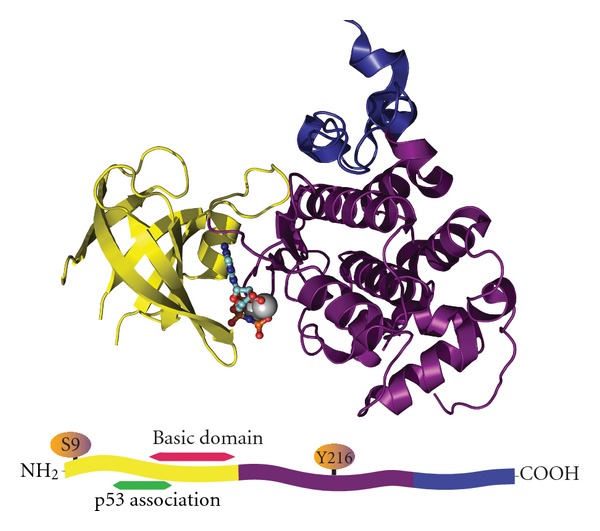
Glycogen synthase kinase-3*β* (GSK-3*β*) structure. GSK-3*β* is a 433 residue protein consisting of 3 distinct structural domains. The N-terminal domain (yellow) consists of the first 134 residues and forms a 7-strand *β*-barrel. A short linker from the N-terminal domain, residues 135–151 connect the N-terminal domain to the *α*-helical domain (magenta). The *α*-helical domain is composed of residues 152–342. Sandwiched between the N-terminal and *α*-helical domain is the ATP-binding site. The C-terminal domain consists of residues 343–433 (blue). A strand diagram of GSK-3*β*. Phosphorylation of Ser-9 inactivates the enzyme, while phosphorylation of Tyr-216 activates. The p53 association region and basic domain region are both located in the N-terminal domain. Image was made using PyMol Molecular Graphics Software version 1.3 with the PDB structure 1UV5.

**Figure 2:**
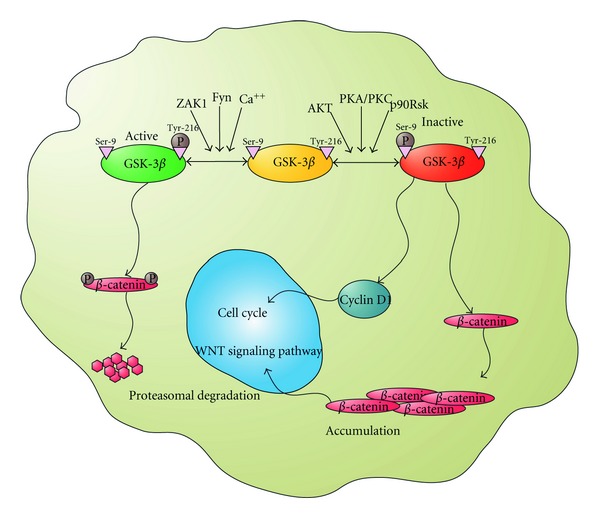
Regulation of GSK-3*β*. GSK-3*β* is a multifunctional kinase that has a role in various signaling pathways that regulate cell fate. ZAK1 or Fyn can phosphorylate Tyr-216 which increases the GSK-3*β* activity. GSK-3*β* can phosphorylate downstream targets like *β*-catenin and degrade it through the ubiquitin-proteasome system. Akt and PKC on the other hand can attenuate GSK-3*β* enzymatic activity by phosphorylating Ser-9. Inhibition of GSK-3*β* activity therefore leads to stabilization and accumulation of *β*-catenin in the cytosol, which is shuttled into the nucleus where it functions to regulate gene expression. GSK-3*β* is also involved in cell cycle regulation through the phosphorylation of cyclin D1, which results in the rapid proteolytic turnover of cyclin D1 protein.

GSK-3*β* is comprised of 12 exons in humans and 11 exons in mice with the ATG start codon located within exon 1 and the TAG stop codon found in the terminal exon. The gene product is a 46 kDa protein consisting of 433 amino acids in the human and 420 amino acids in the mouse.  Figure 1 shows the overall structure of GSK-3*β*. It is similar to other Ser/Thr kinases [10, 11]. The N-terminal domain is comprised of the first 135 residues and forms a 7-strand *β*-barrel motif. A small linker region connects the N-terminal domain to the central *α*-helical domain formed by residues 139 through 342. The ATP-binding site lies at the interface of the N-terminal and *α*-helical domains. Residues 343 through 433 form the C-terminal domain, which is outside of the classical Ser/Thr kinase core fold. These residues form a helix/loop domain that interacts with the core *α*-helical domain. The N-terminal amino acids 78 through 92 are necessary for association with p53 (Figure 1). The activity of GSK-3*β* can be reduced by phosphorylation at Ser-9. Several kinases are able to mediate this modification, including p70S6 kinase, p90RSK, PKC, and Akt [12, 13]. In opposition to the inhibitory phosphorylation of GSK-3*β* at Ser-9, phosphorylation of GSK-3*β* at Tyr-216 by ZAK1 or Fyn increases its enzyme activity [14] (Figure 2).

Dysregulation of GSK-3*β* expression leads to many pathological conditions, including diabetes (or insulin resistance), neuronal dysfunction, Alzheimer's disease [15–18], schizophrenia [19], Dopamine-associated behaviors [20], bipolar disorders [21], Parkinson's disease [22], and cancer. Of special interest is the involvement of GSK-3*β* in cancer with data supporting a role as a tumor suppressor and tumor promoter, a discrepancy that at least in part depends on both cell type and signaling environment. For example, GSK-3*β* has been shown to inhibit androgen receptor-stimulated cell growth in prostate cancer, thus acting as a tumor suppressor [23]. In contrast, GSK-3*β* is highly expressed in colorectal cancer [24, 25] and has been shown to participate in nuclear factor-*κ*B (NF-*κ*B) mediated cell survival in pancreatic cancer [26], thus behaving as a tumor promoter. Moreover, the kinase has dual functions in the regulation of cell survival, where it can either activate or inhibit apoptosis [3, 27], further complicating its involvement in cancer. This paper will focus on how GSK-3*β* can both activate as well as protect from apoptosis with a focus on oncology.

Regulation of *β*-catenin levels is a critical step in Wnt signaling. *β*-Catenin is phosphorylated by GSK-3*β* and then degraded through the ubiquitin-proteasome system [28–30]. Inhibition of GSK-3*β* activity leads to stabilization and accumulation of *β*-catenin in the cytosol, which is shuttled into the nucleus and regulates gene expression (Figure 2). GSK-3*β* is also involved in cell cycle regulation through the phosphorylation of cyclin D1, which results in the rapid proteolytic turnover of cyclin D1 protein [1, 31] (Figure 2). Direct overexpression of wild-type GSK-3*β* is known to induce apoptosis in various cell types in culture, and specific inhibitors of GSK-3*β* are able to stop this apoptotic signaling [6, 7, 9, 32]. The detailed molecular mechanism of GSK-3*β*'s proapoptotic effect is as yet unknown, but it involves regulation of metabolic and signaling proteins, transcription factors, and gene expression [4, 33].

GSK-3*β* is required for proper development [4] and is ubiquitously expressed in the animal kingdom. GSK-3*β* protein was originally isolated from skeletal muscle, but though widely expressed, the protein is most abundant in brain tissue, especially neurons. The high level of expression in brain tissue is likely due to its vital role in neuronal signaling. In neuronal cells, GSK-3*β* is required for dendrite extension and synapse formation in newborns.

## 2. Regulation of Apoptosis by GSK-3**β**

GSK-3*β* has been shown to induce apoptosis in a wide variety of conditions including DNA damage [34], hypoxia [35], endoplasmic reticulum stress [36], and Huntington's disease-associated polyglutamine toxicity [37]. In cell culture studies, apoptosis was either attenuated or fully abrogated by inhibiting GSK-3*β* in primary neurons [38], HT-22 cells [39], PC12 cells [40], and human SH-SY5Y neuroblastoma cells [36, 41].

GSK-3*β* promotes apoptosis by inhibiting prosurvival transcription factors, such as CREB and heat shock factor-1 [42], and facilitating proapoptotic transcription factors such as p53 [34]. A list of some alternative conditions where GSK-3*β* facilitates apoptosis is given in Table 1. A large number of proteins have been shown to interact with the tumor suppressor transcription factor p53 to regulate its actions [43, 44], which has been implicated in the proapoptotic actions of GSK-3*β* in several studies. Following DNA damage, the normally short-lived p53 protein is stabilized and modified by a complex array of posttranslational modifications, such as phosphorylation, acetylation, methylation, ubiquitination, sumoylation, glycosylation, and neddylation. One of these regulatory proteins is GSK-3*β*, which forms a complex with nuclear p53 to promote p53-induced apoptosis [34, 45, 46]. GSK-3*β* binds directly to p53, and the C-terminal region of p53 is necessary for this interaction [45]. GSK-3*β* was shown to directly phosphorylate p53 at Ser-33 [47] and to mediate p53 phosphorylation at Ser-315 and Ser-376 [48, 49]. GSK-3*β* also promotes p53-mediated transcription of specific genes and regulates the intracellular localization of p53 [45, 46, 49]. In addition to GSK-3*β* regulating p53, GSK-3*β* is also regulated by p53. The activity of GSK-3*β* is increased by a phosphorylation-independent mechanism of direct binding of p53 to GSK-3*β* [34]. Nuclear localization of GSK-3*β* may also be regulated by binding of activated p53 [50].

**Figure 3:**
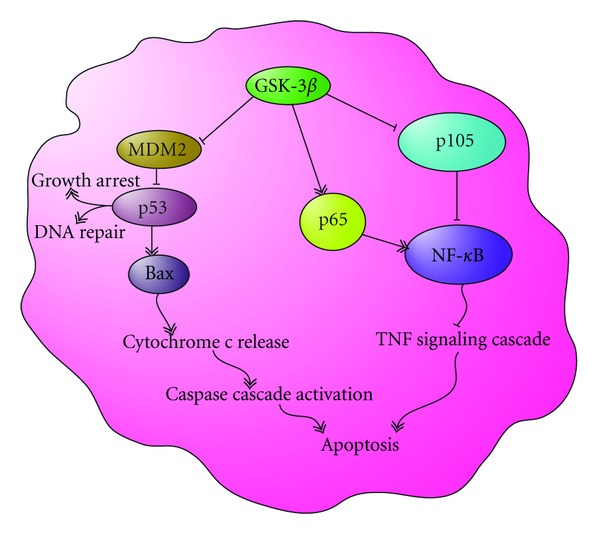
GSK-3*β*'s role in apoptosis signaling. The above schematic shows the role of activated GSK-3*β* and its role in regulating apoptosis. Active GSK-3*β* inhibits MDM2 regulation of p53, leading to DNA repair and growth arrest, and in some cases the activation of the caspase cascade through Bax to promote apoptosis. Active GSK-3*β* also positively regulates NF*κ*B by activating IKK, I*κ*B, and p65, leading to the inhibition of TNF-mediated apoptosis. These actions inhibit the initiation of apoptosis through the TNF signaling cascade.

In addition to direct interaction, GSK-3*β* can regulate p53 levels through the phosphorylation of the p53-specific E3 ubiquitin ligase MDM2 [69]. Regulation of p53 by MDM2 is multifaceted. In the classical model, N-terminal phosphorylation of p53 at Ser-15 (mouse Ser-18) and Ser-20 (mouse Ser-23) inhibits the interaction with MDM2 and thereby prevents MDM2-mediated ubiquitination and the resulting proteasomal degradation of p53 [44] (Figure 3). Stabilized p53 then enters a complex regulatory network to induce DNA binding and transcriptional activation of p53 target genes, in part through the recruitment of coactivators and corepressors. This determines the specific cellular response, which can include survival, growth arrest, DNA repair, or apoptosis [44]. Inhibition of GSK-3*β* in hippocampal neurons protected it from radiation-induced apoptosis [9, 70]. Similar protection from GSK-3*β* inhibition has been seen in primary neurons [38]. The mechanism of protection from radiation-induced apoptosis in these cells involves subcellular localization and interaction of GSK-3*β*, p53, and MDM2. GSK-3*β* inhibition blocks radiation-induced accumulation of p53 by upregulating levels of MDM2 that subsequently result in decreased radiation-dependent apoptosis [71]. In addition to abrogation of radiation-induced p53 phosphorylation, accumulation, and nuclear translocation, GSK-3*β* inhibition results in the accumulation of MDM2 and sequestration of GSK-3*β*, p53, and MDM2 in the cytoplasm where p53 cannot act on its target genes [71]. The role of attenuated p53 function in the prosurvival effects of the GSK-3*β* inhibitors, has also been previously described [34, 46, 70, 72, 73].

In regulation of the apoptotic response, mammalian cells employ multiple prosurvival proteins from the Bcl-2 family (Bcl-2, Bcl-X_L_, Bcl-w, Mcl1, and A1) that antagonize the proapoptotic function of Bax and Bak [34, 74]. Bax and Bak localize to the mitochondrial outer membrane and trigger death signals leading to cytochrome *c* release to the cytosol [74, 75]. Apoptosis requires a group of effector caspases to dismantle the cells. Cytochrome *c* activates caspase-9, which subsequently activates caspase-3 [76]. The activation of caspase-3 is an essential step leading to cleavage of the DNA repair enzyme, poly (ADP-ribose) polymerase (PARP), resulting in genomic DNA fragmentation. Bax protein levels and cleavage (activation) of caspase-3 were increased due to radiation and were abrogated by GSK-3*β* inhibitors [77] (Figure 3). GSK-3*β* was also found to be associated with mitochondrial apoptotic signaling. Inhibition of GSK-3*β* prevented mitochondrial release of cytochrome *c*, which is known to activate caspase-3 and initiate apoptosis [34]. Phosphatidylinositol 3-kinase (PI3-kinase) and its downstream effector, the protein-serine/threonine kinase Akt, a negative regulator of GSK-3*β*, play an important role in preventing apoptosis by blocking activation of the caspase cascade [78].

## 3. Survival-Promoting Effects of GSK-3**β**

GSK-3*β* is involved in multiple signaling pathways and has many phosphorylation targets. It should therefore not be surprising that GSK-3*β* has both pro- and antiapoptotic roles. The overall effect of GSK-3*β* on cell survival varies depending on cell type, transformation status, and the specific signaling pathway being activated. For example, despite evidence for a substantial proapoptotic role of GSK-3*β*, it is the inhibition of GSK-3*β* that promotes apoptosis and decreases viability in neuroblastoma cells [79]. Several examples of pro-survival roles of GSK-3*β* not mentioned here are summarized in Table 2 [80–84].

Additionally, while GSK-3*β* has been typically identified as an activator of p53-mediated apoptosis [34], conflicting reports suggest an inhibitory effect of GSK-3*β* signaling on p53 activation. Inhibition of GSK-3*β* blocks activation of MDM2 by reducing Ser-254 phosphorylation. This prevents p53 degradation and promotes apoptosis despite the induction of p53 ubiquitination. Similarly, ionizing radiation was found to induce an inactivating phosphorylation at Ser-9 of GSK-3*β*, corresponding to hypophosphorylation of MDM2 and accumulation of p53 [69]. In contrast to its proapoptotic effects, this data suggests that GSK-3*β* inhibits apoptosis under basal conditions through MDM2-dependent degradation of p53. Overexpression of *β*-catenin, a downstream signaling factor negatively regulated by GSK-3*β*, was found to increase basal p53 levels by blocking both MDM2-dependent and independent degradation in neuroblastoma cells [85], providing additional supporting evidence for an inhibitory effect of GSK-3*β* on p53-mediated apoptosis. Interestingly, a negative feedback loop exists between *β*-catenin and p53; while *β*-catenin upregulates p53 levels, the activation of p53 results in degradation of *β*-catenin through GSK-3*β* [86]. While the majority of publications suggest a proapoptotic role for GSK-3*β* in p53 signaling, it is clear that more comprehensive studies are needed in order to fully understand the p53-GSK-3*β* relationship.

GSK-3*β* is specifically required for hepatocyte survival in normal embryos, and GSK-3*β* knockout mice are embryonically lethal between E13.15–14.5. Hepatocyte apoptosis in GSK-3*β* knockout mice and mouse embryonic fibroblasts results only after exposure to tumor necrosis factor (TNF), while inhibition of GSK-3*β* in wild-type cells with lithium increases TNF sensitivity. GSK-3*β* loss in these cells has a detrimental effect on the action of NF-*κ*B, which protects against TNF-induced apoptosis [88]. Other studies have shown that GSK-3*β* directly promotes NF-*κ*B stability and activation through both the degradation of p105 and activation of the p65 subunit, suggesting a likely mechanism for lithium-induced TNF hypersensitivity [89, 90] (Figure 3). The role of GSK-3*β* on NF-*κ*B activation may also be mediated indirectly through inhibition of *β*-catenin, as cancer cells with high *β*-catenin levels are especially sensitive to TNF-induced death [91].

Despite the abundance of evidence implicating GSK-3*β* in protection from TNF-mediated apoptosis, a few conflicting reports further complicate our understanding of the pathway. A more recent study claims that GSK-3 inhibition does indeed reduce NF-*κ*B activity but does not result in TNF-mediated apoptosis, potentially due to the activation of pro-survival genes through Wnt signaling [92]. Similarly, TNF sensitization by lithium in multiple sarcoma cell lines was found to be independent of both GSK-3*β* and NF-*κ*B [93] while GSK-3*β* inhibition in prostate cancer and HEK cells actually increased NF-*κ*B activity despite promoting TNF-induced apoptosis [94].

The specifics of apoptosis regulation by GSK-3*β* remain both ambiguous and complex, requiring further research in order to determine the mechanisms of action responsible for differential control of cell survival. In addition to variations in cell signaling and proliferation status, the effect of GSK-3*β* on apoptosis may depend on cellular localization. Only cytosolic GSK-3*β* was found to inhibit TNF-mediated apoptosis [80] while apoptosis enhances nuclear localization [95], suggesting a potential localization-based mechanism for differential apoptotic regulation. Insufficient data is available to explain the contradictory effects proposed for GSK-3*β* on p53-mediated apoptosis, and a more detailed study is required in order to determine the reasons for these observed differences, but differential localization of p53, MDM2, and GSK-3*β* may help define the regulatory role of GSK-3*β* in various systems.

## 4. Positive Regulators of GSK-3**β**

Several molecules are known to potentiate the downstream effects of GSK-3*β* (Table 3). Positive regulators of GSK-3*β* are often utilized for enhancing the proapoptotic effects of GSK-3*β* in the context of chemotherapy for cancer treatment (reviewed in [96]). These regulators typically operate through an indirect mechanism, actually serving as inhibitors for upstream negative regulators. For example, GSK-3*β* activity is increased upon inhibition of PI3-Kinase with wortmannin or LY294002 [97–99]. Many GSK-3*β* regulators act to inhibit Akt by blocking its activation or kinase activity. The kinase inhibitor staurosporine and the COX-2 inhibitor Celecoxib block the activating phosphorylation of Akt by PDK [100–104]. Additionally, curcumin dephosphorylates Akt to prevent its downstream inactivation of GSK-3*β* [102], as does the histone deacetylase inhibitor Trichostatin A, in a PP1-dependent manner [105]. Akt/protein kinase B signaling inhibitor-2 (API-2) appears to suppress both Akt activation and kinase activity independent of any upstream inhibitor effects [106].

Alternative GSK-3*β* regulators have less defined and more indirect mechanisms. The mTOR inhibitor rapamycin has been shown to activate GSK-3*β* with some studies suggesting a potential influence of the mTOR pathway on GSK-3*β* regulation through phosphorylation by s6 kinase [107, 108]. Other molecules target the ability of GSK-3*β* to degrade cyclin D1. Vitamin A derived retinoids and multiple differentiation-inducing factors (DIFs) enhance GSK-3*β* activation and kinase activity [109–112] as a means for cyclin D inhibition to promote cell cycle arrest and differentiation.

## 5. Inhibitors of GSK-3**β**

While a potential therapeutic role of GSK-3*β* inhibitors has been suggested for some time, they have gained significant interest as a clinical tool over the past decade. GSK-3*β* inhibitors are currently being utilized for the treatment of various diseases including Alzheimer's disease [113, 114] and other neurodegenerative diseases [18], diabetes, inflammatory disorders [115], radiation damage, and cancer [116]. Various pharmaceutical companies have these inhibitors in clinical trials [116]. A classical example of a nonspecific GSK-3*β* inhibitor is lithium [21], which has been shown to inhibit GSK-3*β* with an IC_50_ of approximately 2 mM in an uncompetitive manner with respect to peptide substrate. Lithium was found to inhibit GSK-3*β* in a competitive manner by binding directly to magnesium-binding sites of the enzyme [117], thus providing evidence for a molecular mechanism for enzyme inactivation by lithium ions. Four distinct regions of GSK-3*β* have been targeted for inhibition: the Mg^2+^ ATP-binding active site, a separate Mg^2+^-binding site, the substrate-binding groove, and the scaffold-binding region [33, 118]. Several inhibitors compete with Mg^2+^ and/or ATP to occupy its binding site. However, the specificity of these inhibitors towards GSK-3*β* relative to other kinases varies significantly (Table 4). Structural studies have further elucidated molecular mechanisms for substrate selection and GSK3-*β* inhibition [119–125]. Beryllium was shown to compete with both ATP and Mg^2+^, while lithium competed only with Mg^2+^ [126].

The small molecule inhibitors of GSK-3 SB-216763 and SB-415286 are structurally distinct maleimides that inhibit GSK-3*α*/*β* *in vitro*, with K_i_s of 9 nM and 31 nM, respectively, in an ATP competitive manner [127]. Hymenialdisine [128] and paullones [129] also inhibit GSK-3*β* in an ATP competitive manner. Indirubins inhibit GSK-3*β* in an ATP competitive manner with a IC_50_ of 50–100 nM [130–132]. Small molecule inhibitors like TZDZ8 that are thiadiazolidinones inhibit GSK-3*β* with a IC_50_ of 2 *μ*M in a noncompetitive manner [133, 134]. The other type of GSK-3*β* inhibitors is represented by cell-permeable, phosphorylated substrate-competitive peptides which interact with the phospho-recognition motif comprising R96, R180, and K205 to prevent substrate access to the active site. There are also GSK-3*β*-inhibiting peptides that contain GSK-3*β* interacting domains, block the interaction between Axin and GSK-3, and prevent *β*-catenin phosphorylation [135]. In the recent decade small molecule inhibitors of GSK-3*β* are emerging as a promising drug for treatments against neurodegenerative diseases, radiation damage, Alzheimer's disease, diabetes, and cancer [116].

## 6. Exploiting the GSK-3**β** Conundrum

GSK-3*β* signaling is a complex process influenced not only by cellular type and transformation status, but by environmental and cellular conditions. Survival signals have been mainly determined by studies involving GSK-3*β* inhibition, through gene silencing or pharmacologic inhibition. The resulting inhibition of apoptosis is complex, and requires further elucidation. However several studies suggest that the effects may at least in part be mediated by the effect of GSK-3*β* on NF-*κ*B levels. In addition, it is clear that subcellular localization is important, as only cytosolic GSK-3*β* seems to be able to mediate the survival signals.

Notably, the role in promotion of apoptosis by GSK-3*β* has been more clearly delineated. It performs this task by both facilitating proapoptotic signals while inhibiting anti-apoptotic molecules. This signal interplay occurs mostly at the level of the mitochondria, and combined with the association with primarily nuclear GSK-3*β*, suggests a downstream role of GSK-3*β* in modulation.

So how do we exploit these paradoxical roles of GSK-3*β*? In healthy cells, the shift to pro-survival modes is important for cell survival under conditions of cellular stress. In these cases, the upstream signals seem to override the mitochondrial-based apoptotic machinery to allow the cells to escape potentially lethal damage. There have been attempts to exploit these pro-survival roles in neurodegenerative diseases, which are typified by high apoptosis rates. Reduction of disease-associated apoptosis by GSK-3*β* modulating agents can restore balance to off-kilter apoptotic machinery, resulting in decreased cellular turnover and the resultant protection of the at-risk neuronal population. In addition to diabetes, and neurodegenerative disorders, we believe that GSK-3*β* inhibition may play a promising role in patients receiving irradiation.

While radiation dose-escalation has been important for the treatment of multiple cranial tumors (e.g., brain metastases, primary gliomas) and benign disorders (e.g., vestibular schwannoma, meningioma), the treatment is limited by the effects of irradiation on healthy surrounding neurons. It has been demonstrated that GSK-3*β* inhibition can protect hippocampal neurons (in primary culture and murine pups) from irradiation-induced damage [9, 70]. Thotala et al. demonstrated improved survival of intestinal crypt cells and increased latency to murine GI-related death from irradiation [77]. This report suggested that GSK-3*β* inhibitors could reduce deleterious consequences of intestinal irradiation and possibly improve patient quality of life measures. It would be worthwhile to explore their utility in syngenic murine models of neural cancer, murine tumor xenografts, as well as human clinical trials of patients in the setting of re-irradiation (e.g., recurrent glioma). Reports of radiation protection have also been demonstrated with small molecular inhibitors of GSK-3*β* in the gastrointestinal system.

In cancer, however, the apoptotic machinery is often defective allowing cells to undergo unregulated proliferation. In this case, negative regulation of GSK-3*β* can serve to tip the balance in favor of apoptosis. Dickey et al. demonstrated the ability of GSK-3*β* inhibition to effectively enhance cell death of neuroblastoma cells *in vitro *and in a murine xenograft model [79]. Similar findings have been demonstrated in glioma [81, 82]. The interplay between GSK-3*β* regulation and other cell death stimuli is being carefully studied across a wide variety of cancer types, and there is promising data suggesting a strong role for this form of therapy in the near future. The bifunctional role of GSK-3*β* as a facilitator of apoptosis and a mediator of pro-survival signals has important implications in both the generation of novel therapies and the understanding of complex disease states.

The use of both positive and negative regulators of GSK-3*β* offers exciting treatment possibilities for a multitude of diseases. The complexity of the GSK-3*β* network requires careful examination, however, when considering modulating its function in a clinical setting. More studies are required to clearly understand the effects of regulating GSK-3*β* on the multiple signaling pathways involved in growth, development, and metabolism. The effect of GSK-3*β* on cell survival and apoptosis appears to be context dependent, and the required mode of action will likely depend on the specific pathway, cell type, and disease being targeted. While the vast network of GSK-3*β* offers a treatment option for multiple diseases, it also requires careful consideration of all the factors involved in order to prepare against potential side effects.

## Figures and Tables

**Table 1:** Conditions where GSK-3*β* facilitates apoptosis.

System or stimulus	Mechanism
C(2) Ceramide-associated damage	Inhibits the phosphorylation of AKT and ERK pathways and through the dephosphorylation of GSK-3*β* [51]. GSK-3*β* inhibitors have been shown to inhibit apoptosis through inhibiting dephosphorylation of AKT and GSK-3*β* [52].

LPS-mediated endotoxic shock	While specific apoptotic studies have not been performed, LPS has been shown to stabilize apoptotic signal-regulating kinase-1 (ASK-1), a serine-threonine kinase associated with stress-induced apoptosis [53].

Immune system	Regulates in apoptosis of activated T-Cells [54].

HIV-mediated neuronal damage	Inhibits NF-*κ*B [55–57].

Neurodegenerative disease-related toxicity and oxidative stress	Neuronal or oligodendrocyte injury or toxicity (including prion peptide) is associated with increased activity of GSK-3*β*[51, 58–64].
	Negative regulators of GSK-3*β* are associated with increased survival factors [51, 58–64] and neuroprotection [9, 38].

ER stress	ER stress can lead to dephosphorylation of pGSK-3*β*(S9), leading to stress-induced apoptosis through activated caspase-3 [12–14, 26, 28].

Hypoxia/ischemia	Activates mitochondrial death pathway [35, 65–68].

**Table 2:** Other pro survival roles of GSK-3*β*.

System	Mechanism
ER stress	Reduces expression of the proapoptotic transcription factor CHOP/GADD153 [87].
Glioblastoma differentiation	Promotes self-renewal through interaction with Bmi1 [81].
Death receptor complex	Inhibits apoptotic signaling and caspase activation [83].
Chemotherapy	Targeted by death-inducing drugs suggesting an inhibitory role [84].
Oncogenic activation	Inhibits apoptotic activation by c-myc [82].
Glucose metabolism	Prevents apoptosis through mitochondrial stabilization [82].

**Table 3:** List of known positive regulators of GSK-3*β*.

Activator	Activation potency	Mode of activation	Notes
Celecoxib	IC_50_ = 3.5 *μ*M	Inhibits PDK phosphorylation of Akt	COX-2 inhibitor [100].
Staurosporine	IC_50_ = 0.22 *μ*M	Inhibits PDK phosphorylation of Akt	General kinase inhibitor (including PKA/PKC) [101, 103, 104].
Trichostatin A	Unknown	Induces Akt dephosphorylation	HDAC inhibitor acts through PP1 [105].
Curcumin	Unknown	Akt dephosphorylation	Direct target not known [102].
Akt/protein kinase B signaling inhibitor-2 (API-2)	Unknown	Suppresses Akt kinase activity and activation	Does not affect upstream Akt activators [106].
Wortmannin	IC_50_ = 5 nM	Inhibits PI3-Kinase	Indirect effect on GSK-3*β* [97, 98].
LY294002	IC_50_ = 1.4 *μ*M	Inhibits PI3-Kinase	Likely affects ATP binding to kinase [98, 99].
Rapamycin	Unknown	Potentially inhibits S6K1	mTOR pathway can also inhibit GSK3 [107, 108].
Differentiation-inducing factors (DIFs)	Unknown	Enhances GSK-3*β* kinase activity and promotes nuclear localization	Reduces inhibitory phosphorylation and enhances activating phosphorylation [111, 112].
Retinoids	Unknown	Reduces inhibitory phosphorylation of GSK-3*β*	Promotes GSK-3*β*-dependent cyclin D1 degradation [80, 109].

**Table 4:** Selected list of known GSK-3*β* inhibitors.

Inhibitor	Inhibition potency	Mode of inhibition	Notes
Beryllium	IC_50_ = 6 mM	Mg competitor	Also inhibits cdc2
Lithium	K_i_ =2 mM	Mg competitor	
Anilino maleimides (SB216763, SB415286)	K_i_ = 10–30 nM	ATP competitor	Does not inhibit a range of other kinases
Arylpyrazolopyridazines (e.g., 6-aryl pyrazole [3,4-b] pyridine 4)	IC_50_ = 0.8–150 nM	ATP competitor	Also inhibits CDK2
Bisindole maleimides (e.g., Ro 31-8220, GF 109203x)	IC_50_ = 5–170 nM	ATP competitor	Also inhibits PKC
Indirubins (6-bromoindirubin-3′-oxime, aka BIO)	IC_50_ = 5–50 nM	ATP competitor	Also inhibits CDKs
Paullones (alsterpaullone)	IC_50_ = 4–80 nM	ATP competitor	Also inhibits CDKs
Pseudosubstrate peptide	K_i_ = 0.7 mM	Substrate competitor	Specific
